# The Systemic Effects of Exercise on the Systemic Effects of Alzheimer’s Disease

**DOI:** 10.3390/antiox11051028

**Published:** 2022-05-23

**Authors:** Dora Aczel, Bernadett Gyorgy, Peter Bakonyi, RehAn BukhAri, Ricardo Pinho, Istvan Boldogh, Gu Yaodong, Zsolt Radak

**Affiliations:** 1 Research Institute of Sport Science, University of Physical Education, 1123 Budapest, Hungary; aczel.dora555@gamil.com (D.A.); bernike220@gmail.com (B.G.); bakonyi.peti@gmail.com (P.B.); dr.syedrehan@gmail.com (R.B.); 2 Laboratory of Exercise Biochemistry in Health, Graduate Program in Health Sciences, School of Medicine, Pontifícia Universidade Católica do Paraná, Curitiba 80215-901, Brazil; rapinho12@gmail.com; 3 Department of Microbiology and Immunology, University of Texas Medical Branch at Galveston, Galveston, TX 77555, USA; sboldogh@utmb.edu; 4 Faculty of Sports Science, Ningbo University, Ningbo 315211, China; guyaodong@hotmail.com; 5 Faculty of Sport Sciences, Waseda University, Tokorozawa 359-1192, Japan

**Keywords:** Alzheimer’s disease, amyloid-β, exercise, metabolism, peripheral organs, free radicals

## Abstract

Alzheimer’s disease (AD) is a progressive degenerative disorder and a leading cause of dementia in the elderly. The etiology of AD is multifactorial, including an increased oxidative state, deposition of amyloid plaques, and neurofibrillary tangles of the tau protein. The formation of amyloid plaques is considered one of the first signs of the illness, but only in the central nervous system (CNS). Interestingly, results indicate that AD is not just localized in the brain but is also found in organs distant from the brain, such as the cardiovascular system, gut microbiome, liver, testes, and kidney. These observations make AD a complex systemic disorder. Still, no effective medications have been found, but regular physical activity has been considered to have a positive impact on this challenging disease. While several articles have been published on the benefits of physical activity on AD development in the CNS, its peripheral effects have not been discussed in detail. The provocative question arising is the following: is it possible that the beneficial effects of regular exercise on AD are due to the systemic impact of training, rather than just the effects of exercise on the brain? If so, does this mean that the level of fitness of these peripheral organs can directly or indirectly influence the incidence or progress of AD? Therefore, the present paper aims to summarize the systemic effects of both regular exercise and AD and point out how common exercise-induced adaptation via peripheral organs can decrease the incidence of AD or attenuate the progress of AD.

## 1. Introduction

Alzheimer’s disease (AD) is known as a progressive degenerative disorder of the central nervous system (CNS), characterized by neuronal dysfunctions and changes in brain structure and function [1,2,3]. AD is the most common irreversible cause of dementia in elderly patients, estimated to comprise 60–80% of cases [1,4]. Pathophysiology of AD includes the oxidative state, the aggregation of pathological amyloid-β (Aβ), and abnormal accumulation of hyperphosphorylated neurofibrillary tangles of the tau protein, followed by inflammation in the central cortex and limbic system of the brain, which results in neuronal atrophy and a loss of synapses [5,6]. While amyloidogenesis is a well-controlled process in healthy tissues, pathologic amyloid plaques can accumulate in various tissues in AD. The formation of amyloid plaques is considered the first sign of the illness, not only in the CNS but also in peripheral organs, resulting in a systemic disorder [7,8,9]. Available evidence supports the belief that the decreased clearance of Aβ is one of the key processes in AD’s pathomechanism [10]. The most common early clinical symptom of amyloid plaque accumulation is difficulty remembering recent events [11]. Furthermore, longitudinal studies have shown that depressive symptoms that occur more than ten years before the onset of AD are associated with mild cognitive impairment [12] and memory complaints [13]. Physical disability continues to decline in cognitive functions and behavioral and social skills. AD reduces functioning mobility, lower quality of life conditions, and higher dependence on other people [11]. Due to its increased prevalence and incurability, AD is one of the major priorities of the healthcare system and one of the most significant social and economic challenges in modern society (World Health Organization (WHO) and Alzheimer’s Disease International (ADI), 2012). In addition, several other common disorders often appear with AD, such as diabetes [14], vascular abnormalities [15], and inflammation [16].

The dependence of AD on environmental and lifestyle factors is noted by the close relationship between AD and DNA methylation (DNAm)-based biomarkers, such as PhenoAge. DNAm PhenoAge is a powerful epigenetic biomarker that predicts various aging outcomes, including all-cause mortality, cancer, health, physical activity, and AD. DNAm PhenoAge is based on complex clinical measures of phenotypic age that record differences in lifespan and healthspan. The association between pathologically diagnosed AD and DNAm PhenoAge suggests that those diagnosed with AD present with more than the one-year-older dorsolateral prefrontal cortex than same-aged individuals who were not diagnosed with the disease. Interestingly, age-adjusted DNAm is positively associated with neuropathological hallmarks of AD, such as amyloid load, neuritic plaques, and neurofibrillary tangles [17].

Many different signaling pathway molecules have been investigated at the cellular level as possible targets in AD treatment. One of these promising agents could be pituitary-adenylate-cyclase-activating polypeptide (PACAP), a member of the vasoactive intestinal polypeptide (VIP)—a secretin-growth-hormone-releasing hormone (GHRH)—glucagon superfamily [18]. PACAP, due to its oxidative-stress-reducing effects [19], has a protective impact against aging [20,21], amyloidosis [21], and neurodegenerative diseases. For example, in AD, correlating with accumulating amounts of Aβ, decreased PACAP signaling activity has been observed [21]. The preventive influence of PACAP on AD has been reported to protect against Aβ toxicity and attenuate AD’s severity in animal models [18,22]. PACAP neuropeptide has three G-protein-coupled seven-transmembrane receptors: pituitary-adenylate-cyclase-activating polypeptide type 1 receptor (PAC1R), binds PACAP with the highest affinity, and vasoactive intestinal peptide receptors (VPAC1R and VPAC2R) bind PACAP with lower binding affinity. All three receptors have been detected in CNS and in many peripheral tissues. PACAP can activate intracellular messengers such as adenylate cyclase, cyclic adenosine monophosphate (cAMP)/protein kinase A (PKA), the more active, phosphorylated form of PKA (P-PKA), and protein phosphatase 2A (PP2A) [23,24]. These signaling pathways were reported in CNS and suggested to lead to deterioration of neural function, leading to AD’s well-known symptoms in CNS. However, recent observations indicate that this AD-associated cellular signaling pathway is also present in peripheral organs.

## 2. AD and Exercise

Great efforts have been made to find reliable prevention methods and develop drugs or disease-modifying therapies to treat AD, yet without success [25,26,27,28]. These findings emphasize the importance of prevention. It is known that genes can predispose to AD [29,30], but it is also known that lifestyle habits are associated with the incidence of AD [31,32]. It has been reported that physical inactivity increases the incidence of AD [28]. Therefore, it is not surprising that physical activity can reduce the occurrence of AD [32]. Regular exercise has been shown to induce adaptation in the whole body, including the neuronal, cardiovascular, skeletal, immune, digestive, and reproductive systems [33,34]. Furthermore, training in AD (TAD) is known to have a positive impact on alteration of neurotrophin synthesis, attenuation of oxidative stress, inflammation, induction of amyloid-β (Aβ) degrading enzymes, an increase in vascularization and blood flow, and energy metabolism of the brain [32]. It is also known that exercise can reduce the concentration of Aβ in plasma [35] and can be protective against Aβ neurotoxicity with the disease [11].

## 3. Exercise and Peripheral Organs with AD

Physical exercise is recommended as a nonpharmacological method for preventing cognitive decline [36]. Training has antioxidant [37] and anti-inflammatory effects that contribute to neuroprotection and cognitive improvements seen in animal models and AD patients [32,38]. AD is also regarded as a metabolic disorder and most of the attention has been paid to brain metabolism [39]. Interestingly, results indicate that AD is localized in the brain and organs distant from the brain. The cardiovascular system, gut microbiome, liver, testes, and kidney are also affected [40,41,42]. AD may also affect metabolism, such as overproduction and accumulation of pathological amyloid plaques in peripheral organs [32,43,44,45]. These observations make AD a complex systemic disorder [16,46]. In this paper, therefore, we have examined metabolic changes in the cardiovascular system, gut microbiome, liver, testes, and kidney of the amyloid precursor protein/presenilin 1 (APP/PS1) mice [47,48,49,50]. Metabolomic results reveal that the liver was one of the earliest affected organs in APP/PS1 mice during amyloid pathology progression, followed by the kidney and heart [51]. Interestingly, the CNS-related symptoms of AD, such as loss of memory, are readily recognizable, while impaired functions in the liver or kidney are less prevalent.

Several articles have been published on the benefits of physical activity in developing AD in CNS [11], but the peripheral effects have not been discussed in detail. Regular training is known to reduce Aβ biomarker concentrations in the blood and cerebrospinal fluid [52], but only a few data are available to elucidate the peripheral mechanisms. It is interesting to examine the effect of mobility because there may be potential positive systemic effects and consequences on different tissue in AD [32].

The provocative question that arises is the following: is it possible that the beneficial effects of regular exercise on AD are due to the systemic effects of training, rather than only the impact of exercise on the brain? If so, does this mean that the level of fitness of peripheral organs can directly or indirectly influence the incidence or progress of AD? Therefore, the present paper aims to summarize the systemic effects of both regular exercise and AD and point out how common exercise-induced adaptation via peripheral organs can decrease the incidence of AD or attenuate the progress of AD.

## 4. Exercise and the Cardiovascular System with AD

With the progress of the availability of genetic screening, accumulating evidence suggests a possible link between AD-associated allele variants of Apo E4, presenilin 1–2, and heart failure [53,54,55]. However, a recent Mendelian randomization study did not find causal relationships between AD and heart failure in a European population [56]. One of the reasons behind heart failure and cardiac events related to mortalities is due to greater left ventricular (lv) diameter and lv mass [57]. The results of a longitudinal study on middle-aged American aboriginals revealed a 25 gm increase in lv mass in mid-life, which was independent of physical activity and was associated with lower hippocampal volume and higher white matter grade in older life [58]. Moreover, lv hypertrophy was independently related to cortical thickness, pointing out the relationship between lv hypertrophy with adverse changes in brain structure [59].

Metabolic syndrome does not just increase the risk of cardiovascular diseases, including heart failure, but also increases the incidence of AD [60]. The organism’s metabolism level and the organ itself seems to play an important role in the aging process. AD is characterized by metabolic impairment in CNS [32] and the cardiovascular system [61], and has been proposed to be one of the reasons for AD-induced cardiovascular disorders [62]. It has been demonstrated in an animal model of AD that transgenic mice expressing five mutations in human AβPP and PS1 (5XFAD) showed decreased cardiac contractility and mitochondrial functions compared with wild mice. Increased activation of AMP-activated protein kinase (AMPK) levels in the lv and inflammatory markers were also observed in 5XFAD mice [63].

Exercise, especially endurance exercise, has a powerful effect on the cardiovascular system [64]. Molecular mechanisms associated with the cardiovascular system and pathologies of AD have many common pathways. These could be linked to the increased neuronal and myocardial deaths observed in the mixed etiology. Indeed, decreased blood flow and oxygen supply have been reported prior to Aβ deposition in the brain of humans and mouse models and have been suggested to play a role in AD pathology [65,66]. Endothelial nitric oxide (NO) was first recognized as a major vasodilator involved in cardiovascular homeostasis, regulation of blood pressure, and local blood flow [67]. Exercise also stimulates NO release from endothelial cells [68]. This finding may suggest an association between endothelial NO, training, cerebrovascular, and cognition functions in AD. Previous research has recognized NO’s role in causing excessive amyloidogenic processing of APP and elevated local concentrations of Aβ, thus influencing the functional state of microglia and cognitive functions [65]. NO is produced by constitutively active endothelial nitric oxide synthase (eNOS). In addition, increased expressions of APP and β-site APP cleavage enzyme 1 (BACE1) and increased production of Aβ peptides in the cerebral tissue of eNOS-deficient mice were connected to AD [69].

## 5. Exercise and Gut Microbiome in AD

Gastrointestinal comorbidities are prevalent in AD [70], but relatively few studies emphasize the connection between exercise and gut microbiota in AD. This complex mass of microbiota [71,72] contributes to several functions through neuronal, immune, endocrine, and metabolic pathways [73,74]. The genomes of all microorganisms can be helpful, but they are also potentially harmful to the human body. Unfortunately, this large number of microorganisms can influence the development of various diseases outside the digestive system. It has been proven that specific microbiome alterations are associated with cognitive impairment and deficiencies in synaptogenesis [75]. Furthermore, accumulated evidence suggests that the microbiota–gut–brain axis affects neurodegenerative disorders, including dementia and AD [76,77,78]. The microbiome affects both disease progression and cognitive abilities in AD [79,80,81]. Vice versa, AD causes changes in the microbiome, mainly due to the deposition of Aβ plaque, which is associated with the inflammatory response [82]. Short-chain fatty acids (SCFAs) may be involved in these changes: for example, butyrate plays an essential role in suppressing inflammation, increases intestinal mucin production, and affects the expression of tight-junction proteins, which may cause enhanced intestinal permeability [83,84,85].

AD treated with probiotics resulted in changes in the microbiome environment and increased brain function [86]. Physical activity is also a well-known cause of alterations in the microbiome’s composition, even in AD [82,87,88,89,90,91]. A group of AD transgenic mice APP/SP1 which received exercise and probiotic treatment significantly outperformed all other groups in terms of cognitive function, such as the spatial memory, assessed by the Morris Maze Test [82]. Training with probiotic supplementation also altered the level of different bacterial genera in the gut microbiome in AD transgenic mice. Changes were observed mainly in the genera of Bacteroides, Clostridia, Eubacteria, Lactobacillus, Prevotella, and Roseburia (Table 1) [82]. Some of these microorganisms are involved in butyrogenesis, such as Prevotella, Bacteroides, and Lactobacillus [92]. Their abundance increased in trained groups. Furthermore, the level of Lactobacillus johnsonii was positively correlated with Aβ content and covered area in the hippocampus. Elevated levels of L. reuteri were also observed due to training [82]. L. reuteri is known as a vitamin B12 producer, which is obligatory for brain health and low levels of vitamin B12, and is found to be associated with an increased risk of AD [93,94,95]. These observations suggest that regular exercise and nutritional interventions, such as probiotic supplementation, are being proposed to reduce the incidence and decrease the progression of AD [70,96,97].

## 6. Exercise and Liver with AD

Increasing evidence suggests an important role of liver function in the pathophysiology of AD. Proteins, such as amyloid-β and hyperphosphorylated tau, are vital contributors to the onset or progression of AD. The liver is theoretically involved in the peripheral clearance of circulating Aβ in the blood [98].

In addition to the beneficial systemic effects, exercise positively impacts liver function in AD. For example, abnormal levels of liver enzymes associated with the diagnosis of Alzheimer’s and correlated with poor memory and thinking scores have been observed [99]. Furthermore, microbial changes caused by exercise and probiotic treatment alter liver metabolism, mitochondrial content, and antioxidant capacity in APP/PS1 transgenic mice. Training and probiotic supplementation did not significantly raise mitochondrial counts in the AD animal’s liver, based on cytochrome c oxidase subunit 4 (COX4) and peroxisome-proliferator-activated receptor–gamma coactivator 1 alpha (PGC-1α) protein levels. However, on the other hand, the mitochondrial antioxidant capacity changed positively due to regular exercise. Antioxidant signaling proteins, such as nuclear factor erythroid 2-related factor 2 (NRF-2) and superoxide dismutase 2 (SOD2), were also investigated. NRF-2, the major regulator of antioxidant protection, showed an increase in the group of trained and probiotic supplemented animals compared with the AD group. Furthermore, decreased superoxide SOD2 levels were observed in AD, while training prevented this alteration [47].

## 7. Exercise and Gonads with AD

Testes are also peripheral organs affected by AD. In AD organs, decreased numbers of spermatogonia, spermatocytes, and interstitial Leydig cells have been observed. Immunoreactivity of collagen type IV in seminiferous tubules’ basement membrane (bm) was hardly detectable, and decreased bm thickness in AD testes, resulting in changes in blood–testes barrier function.

Studies show that testicular degradation can be compensated for by regular physical activity in a mouse model of AD [100]. In TAD animals, the number of convoluted seminiferous tubules’ cells was partially recovered, and the number of Leydig cells was elevated after physical activity. As a result of training, the thickness of the basement membrane became almost as thick as in WT mice. Expression of the type IV collagen molecule was also elevated, maintaining the integrity of the basal membrane, and thus compensating for the adverse effects of AD [48].

The PACAP, as mentioned above, is expressed in not only the CNS but also peripheral organs, with the highest level in testes [101]. PACAP regulation seems essential for maintaining the typical structure of testes and spermatogenesis and male sex hormone production [102]. It is proven that a lack of PACAP protein or a mistake in PACAP downstream signaling causes morphological and functional changes in testis [21]. The messenger RNA (mRNA) expression and the protein level of the PAC1R were decreased in AD animals compared with WT [48]. Interestingly, this reduction in PAC1R has also been demonstrated in the CNS of AD models [18]. In contrast, the mRNA and protein expression of VPAC1R and VPAC2R did not show a significant difference in AD. In the TAD experimental group, an increase in PAC1R and VPAC1R was noted, whereas a decrease in VPAC2 receptor protein expression was observed. The downstream PACAP signaling pathway elements, such as the cAMP, PKA, P-PKA, and PP2A protein expressions, were also significantly decreased in the samples of AD mice. In TAD testes, expression of the PKA protein was elevated almost to WT levels, and P-PKA and PPA2 were augmented considerably by physical activity [48].

There are few data available about ovary in AD. The most common metabolic disorder in premenopausal women is polycystic ovary syndrome (PCOS) [103]. Patients with PCOS have increased LH-FSH levels, decreased vitamin D and insulin resistance, and obesity [104]. These are important factors also in AD and may increase the risk of the disease [105]. Moderate exercise (guidelines for PCOS suggest at least 150 min of physical activity per week) is helpful in PCOS, so it can contribute to minimize the chance of developing AD [106]. Furthermore, women with premature menopause have an increased risk of AD [107]. Menopausal hormone therapy supplemented with regular training may give the chance to reduce the risk of AD in later life [108,109].

## 8. Exercise and Kidney in AD

Pathological Aβ accumulation has also been observed in kidneys of AD animals leading to possible fibrosis, causing filtration disorders and renal insufficiency [44]. In AD mice, homogenous eosinophilic deposits in the tubular systems and strong Aβ positivity were visible in the kidneys [49].

Physical exercise has been shown to positively affect the morphology and function of kidneys in the AD mouse model. Training can reduce kidney fibrosis, which induces Aβ clearance and may help inhibit the disease’s progression [110]. First of all, exercise reduced Aβ accumulation and diminished eosinophilic deposits [49]. After physical activity in AD mice, the amount of interstitial collagen type I was reduced compared with the untrained group. Furthermore, both normalized mRNA and protein expression of collagen type I were measured in TAD animals [50]. Additionally, as a result of exercise, the immunopositivity and (mRNA and protein) the expression of collagen type IV were normalized/elevated in trained animals [49]. The normalized basement membrane formation can play a role in Aβ elimination via kidneys and help inhibit the disease’s progression [49,110]. Accordingly, collagen type IV has been observed to inhibit Aβ plaque formation [111].

Pathological fibrosis in the kidneys of AD animals raises the role of transforming the growth factor β (TGFβ) pathway [112,113]. TGFβ is an essential factor in the pathogenesis of AD in the brain and a master regulator of renal inflammation and fibrosis, consequently responsible for appropriate filtration [114,115]. TGFβ causes increased collagen expression and accumulation [116]. Physical activity, through TGFβ signalization, may prevent renal fibrosis and support Aβ clearance in the periphery. Activation of canonical and non-canonical TGFβ pathways was observed in AD, which was normalized in TAD mice. Although TGFβ1′s mRNA and protein expression did not significantly differ between WT and AD kidneys, increased expressions were found in TAD samples. As for the receptors, the expression of TGFβRI protein was reduced in AD mice and normalized after physical activity, while the expression of mRNA and protein of TGFβRII changed to the contrary [50].

TGFβ signaling interacts with the group of mitogen-activated protein kinases (MAPKs), such as extracellular signal-regulated kinase 1/2 (ERK1/2), p38 mitogen-activated protein kinase (p38), and Jun N-terminal kinase (JNK) [117]. Members of the MAPK family strongly support distinct functions in the pathogenesis of renal fibrosis. For example, ERK contributes to renal fibrotic transformation, whereas inhibition of ERK activity reduces interstitial fibrosis in AD [118]. The MAPK family shows alteration in the kidney of AD after long-term training. After physical activity, standard ERK and a more active form of the kinase, phospho-ERK expression, and normalized renal function were detected in TAD samples [50]. The protein expression of ERK1/2 kinase was reduced in the kidneys of AD mice, with normalization after physical exercise. The amount of the phosphorylated ERK was significantly elevated in AD samples and showed a significant decrease in TAD mice. The other member of the MAPK family is the p38 protein. Its expression was increased in AD mice, inducing fibrosis, while the phosphorylated form of p38 changed to the contrary. Exercise normalized p38 levels suggest a balancing function of training in p38-mediated fibrosis formation [50,119]. Therefore, inhibition of p38 may be a good target in fibrosis treatment [120]. Furthermore, JNK kinase also affects glomerular filtration [121], and the inhibition of the kinase may suppress interstitial fibrosis [122]. The protein expression of the two isoforms of JNK revealed a significant reduction in AD mice but showed elevation in TAD animals [50].

Matrix metalloproteinase 9 (MMP9) is also involved in fibrotic processes by degrading extracellular matrix elements such as type I, IV, and type V of collagen and other extracellular matrix proteins [123,124]. MMP9 is regulated by TGF signaling in the kidney, and its activation is p38-dependent [123,125]. Surprisingly, a significant increase in MMP9 protein expression was observed in the kidneys of AD mice, but MMP9 expression was dramatically increased after exercise, congruent with the marked decrease in collagen type I in the tubular system [50].

The TGF signaling pathway’s activation can also play an important role in AD pathogenesis through cell cycle/cellular proliferation and apoptosis [126]. Cell proliferation markers, such as cyclin-dependent kinase inhibitor 1 (CDKN1/p21) and proliferating cell nuclear antigen (PCNA) expressions, were measured in AD: CDKN1/p21 activation and substantial PCNA reductions in the tubular system of AD mice were normalized by physical activity [49]. The CDKN1/p21 elevation suggests a cell cycle arrest in AD, and CDKN1/p21 interaction with PCNA can block cells in the S-phase of the cell cycle [127]. Caspase activation can mediate via Aβ accumulation: the more active, cleaved form of caspase 3 appeared in AD samples, indicating increased apoptosis and the loss of tubular cell function, while physical activity reduced its expression [49,128].

The abovementioned neuroprotective PACAP also has a nephroprotective role in various renal pathologies [129]. PACAP is produced in kidneys, where the most dramatic amyloid deposition was found in PACAP knockout mice. This signal molecule may be one of the most promising targets for the renal elimination of Aβ [21]. Examining molecular processes in AD mice, the protein expressions of PAC1R, VPAC1R, and VPAC2R were demonstrated in WT kidneys. In contrast, the expressions of all PACAP receptors were almost undetectable or significantly decreased in AD samples. Interestingly, the level of all PACAP receptors increased in TAD mice: most dominantly, expressions of PAC1R and VPAC1R were elevated [49]. PACAP equally binds both PAC1R and VPAC1R in kidney diseases [130]. PACAP signaling pathway can be regulated through PKA [101]. In AD animals, reduced PKA protein expression was observed, almost normalized after physical activity [49]. The activation/phosphorylation of PKA increases the level of transcription factor cAMP-response-element-binding protein (CREB) [131], the protein level of which was also decreased (or its activated form was undetectable) in AD mice and augmented after exercise [49]. These data suggest PACAP and PKA signaling may be involved in “physical-activity-mediated defense mechanisms” in AD. Furthermore, PACAP also plays an essential role in bone morphogenetic protein (BMP) signaling [132], as does a member of the TGFβ superfamily [133]. Initially, BMP was identified as “only” an osteogenic factor, but nowadays, it is known that BMPs perform several other functions [133,134,135,136]. PACAP and BMP signaling is involved in preventing diseases through physical activity. For example, modified BMP/Smad signaling has been reported in AD mice [49,130]. BMPs and Smad transcription factors regulate the expression of genes associated with fibrosis and basement membrane components, such as collagen [137,138]. Both BMP receptor type 1 (BMPR1) mRNA and protein levels and the expression of bone morphogenetic protein 4 (BMP4), which induces Smad1, showed a noticeable reduction in AD samples. Still, they were significantly augmented as a result of training. Smad2 and Smad3 expressions were also altered in the kidneys of AD mice: Smad2 increased, while Smad3 showed a moderate decrease in AD samples, but physical activity can compensate for these alterations [49].

**Figure 1:**
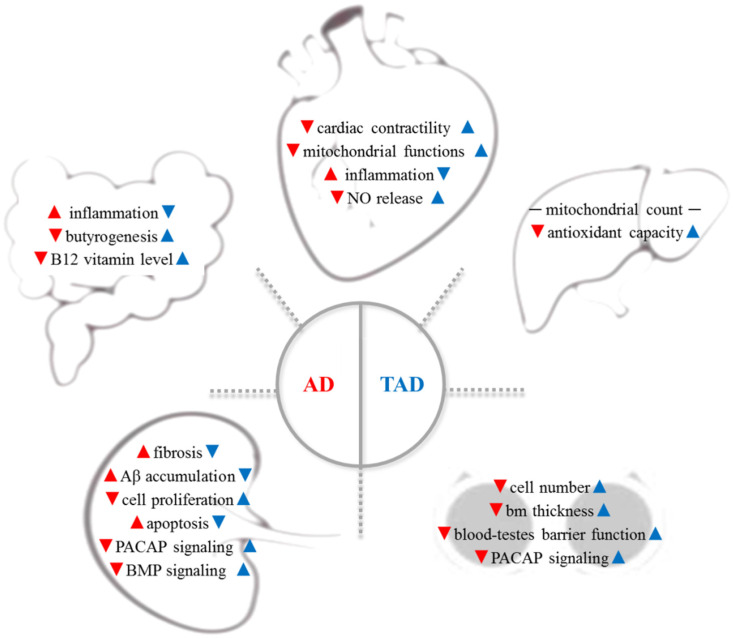
AD is a complex systemic disorder, which induces the degenerative process in organs distant from the brain: cardiovascular system, gut microbiome, liver, testes, and kidney are involved (red arrows). Therefore, increased physical activity has been reported to have a preventive effect on all organs in AD (blue arrows). Aβ—β-amyloid; AD—Alzheimer’s disease; bm—basement membrane; BMP—bone morphogenetic protein; NO—nitric oxide, PACAP—pituitary--cyclase-activating polypeptide; TAD—trained AD.

TGFβRII, ERK, p38, JNK, and Smad phosphorylation occur on serine (Ser) amino acid, which raises a possible activation of serine–threonine protein phosphatases (Ser/Thr phosphatases) in AD [139]. Altered PP2A and PP2B expressions and modified protein phosphorylations have been reported in AD [140,141,142]. PP2A can regulate tau phosphorylation with a higher affinity than PP2B [143]. Reduced PP2A, but an increased PP2B expression in kidneys of AD mice was observed, similarly to the CNS, which can be compensated by physical exercise [49]. Increased phosphorylation on Ser residues, as the result of PP2A expression decrease, can induce the hyperphosphorylation of tau protein or APP, as was predicted in AD. In addition, ERK kinase dephosphorylation can happen dominantly through PP2A activation [144]. Subsequently, reduced expression of PP2A can lead to increased ERK phosphorylation in AD [49]. The PP2B can directly regulate the dephosphorylation of JNK and p38 kinases [145]. Therefore, the decreased activation of these kinases is the consequence of elevated PP2B expression in AD (Figure 1).

## 9. The Effects of Exercise on Peripheral Organs in Alzheimer’s Disease

AD is a complex systemic disorder, which induces the degenerative process in organs distant from the brain: cardiovascular system, gut microbiome, liver, testes, and kidney are involved (Figure 1, red arrows). Therefore, increased physical activity has been reported to have a preventive effect on all organs in AD (Figure 1, blue arrows). 

## 10. Conclusions

These results further support the hypothesis that AD induces the degenerative process of peripheral organs, showing that AD is a systemic disease. Increased physical activity has been reported to have a preventive and debilitating effect on all organs, emphasizing the potential preventive role of regular exercise in AD. The results further suggest that exercise can also attenuate the progress of AD. However, it is unknown how the involvement of different organs in the pathology of AD affects the progress of neurodegeneration. It is suggested here that the systemic nature of the exercise is one of the most powerful natural tools to fight AD.

## Figures and Tables

**Table 1:** Investigation of the systemic effects of exercise on peripheral organs in AD.

	Changes	AD Mice	TAD Mice	Decisive Study
Cardivascular system	Cardiac contractility	↓	↑	
	Mitochondrial functions	↓	↑	
	Inflammation	↑	↓	
	NO release	↓	↑	
Gut Microbiome	Inflammation	↑	↓	Abraham et al. [82]
	Butyrogenesis	↓	↑	
	B12 vitamin level	↓	↑	
	Bacteroides	↓	↑	
	Lactobacillus	↓	↑	
	Prevotella	↓	↑	
Liver	Mitochondrial antioxidant capacity	↓	↑	Téglás et al. [47]
	NRF-2	↓	↑	
	SOD2	↓	↑	
Gonades (testes)	Cell numbers			Szegeczki et al. [48]
	Spermatogonia count	↓	↑	
	Spermatocytes count	↓	↑	
	Leydig cells count	↓	↑	
	Collagen type IV	↓	↑	
	Basement membrane thickness	↓	↑	
	Blood-testes barrier function	↓	↑	
	PACAP signaling			
	PAC1R	↓	↑	
	VPAC1R	-	↑	
	VPAC2R	-	↓	
	cAMP	↓	-	
	PKA	↓	↑	
	P-PKA	↓	↑	
	PP2A	↓	↑	
Kidney	Aβ accumulation	↑	↓	Perényi et al. [49]
	Basement membrane formation			
	Collagen type IV	↓	↑	
	Fibrosis			
	Collagen type I	↑	↓	
	TGFβ1	-	↑	
	TGFβRI	↓	↑	
	TGFβRII	↑	↓	
	ERK1/2	↓	↑	
	Phospho ERK1/2	↑	↓	
	p38	↑	↓	
	phospho 38	↓	↑	
	JNK	↓	↑	
	MMP9	↑	↑↑	
	Cell proliferation			
	CDKN1/p21	↑	↓	
	PCNA	↓	↑	
	Apoptosis Cleaved caspase 3	↑	↓	
	PACAP signaling			
	PAC1R	↓↓	↑↑	
	VPAC1R	↓↓	↑↑	
	VPAC2R	↓	↑	
	PKA	↓	↑	
	CREB	↓	-	
	Phospho CBEB	↓↓	↑	
	BMP1R	↓	↑	
	BMP4	↓	↑	
	Smad1	↓	↑	
	Smad2	↑	↓	
	Smad3	↓	↑	
	PP2A	↓	↑	
	PP2B	↑	↓	

## Data Availability

Not applicable.

## Abbreviations

Aβ—β-amyloid; AD—Alzheimer’s disease; BMP4—bone morphogenetic protein 4; BMP1R—bone morphogenetic protein receptor, type 1; cAMP—cyclic adenosine monophosphate protein kinase A; CREB—cAMP-response-element-binding protein; CDKN1/p21—cyclin-dependent kinase inhibitor 1; ERK1/2—extracellular-signal-regulated kinase 1/2; JNK—c-Jun N-terminal kinase; MMP9—matrix metalloproteinase 9; NO—nitric oxide; NRF-2—nuclear factor erythroid 2-related factor 2; p38—p38-mitogen-activated protein kinase; PAC1R—pituitary-adenylate-cyclase-activating polypeptide type 1 receptor; PACAP—pituitary-adenylate-cyclase-activating polypeptide; PCNA—proliferating cell nuclear antigen; PKA—protein kinase A; P-PKA—phosphoprotein kinase A; PP2A—protein phosphatase 2 A; PP2B—protein phosphatase 2 B; SOD2—superoxide dismutase 2; TAD—trained AD; TGFβ1—transforming growth factor β1; TGFβRI—transforming growth factor β receptor I; TGFβRII—transforming growth factor β receptor II; VPAC1R—vasoactive intestinal peptide receptor type 1; VPAC2R—vasoactive intestinal peptide receptor type 2.
